# GRADE equity guidelines 3: considering health equity in GRADE guideline development: rating the certainty of synthesized evidence

**DOI:** 10.1016/j.jclinepi.2017.01.015

**Published:** 2017-10

**Authors:** Vivian A. Welch, Elie A. Akl, Kevin Pottie, Mohammed T. Ansari, Matthias Briel, Robin Christensen, Antonio Dans, Leonila Dans, Javier Eslava-Schmalbach, Gordon Guyatt, Monica Hultcrantz, Janet Jull, Srinivasa Vittal Katikireddi, Eddy Lang, Elizabeth Matovinovic, Joerg J. Meerpohl, Rachael L. Morton, Annhild Mosdol, M. Hassan Murad, Jennifer Petkovic, Holger Schünemann, Ravi Sharaf, Bev Shea, Jasvinder A. Singh, Ivan Solà, Roger Stanev, Airton Stein, Lehana Thabaneii, Thomy Tonia, Mario Tristan, Sigurd Vitols, Joseph Watine, Peter Tugwell

**Affiliations:** a Bruyère Research Institute, University of Ottawa and Bruyère Continuing Care, 85 Primrose Ave, Ottawa K1R 7G5, Canada; b Department of Internal Medicine, American University of Beirut, P.O. Box: 11-0236, Riad-El-Solh Beirut, 1107 2020 Beirut, Lebanon; c Department of Health Research Methods, Evidence, and Impact (HEI), McMaster University, 1280 Main Street West, Hamilton, ON L8S 4K1, Canada; d Departments of Family Medicine and Epidemiology and Community Medicine, Bruyère Research Institute, University of Ottawa, WHO and EU ECDC Consultant; e School of Epidemiology, Public Health and Preventive Medicine, Faculty of Medicine, University of Ottawa, Room 101, 600 Peter Morand Crescent, Ottawa ON K1G 5Z3 Canada; f Department of Clinical Research, Basel Institute for Clinical Epidemiology and Biostatistics, University of Basel, Spitalstrasse 12, Basel 4031, Switzerland; g Department of Health Research Methods, Evidence and Impact, McMaster University Health Sciences Centre, 1280 Main Street West, Hamilton, Ontario, L8S 4K1, Canada; h Musculoskeletal Statistics Unit, The Parker Institute, Bispebjerg and Frederiksberg Hospital, Copenhagen, Denmark; i Department of Medicine, University of the Philippines-Manila, Manila 1000, Philippines; j Department of Pediatrics, University of the Philippines-Manila, Taft Ave, Manila 1000, Philippines; k Group of Equity in Health, Hospital Universitario Nacional de Colombia, Faculty of Medicine, Universidad Nacional de Colombia; Technology Development Center, Sociedad Colombiana de Anestesiologia y Reanimacion -S.C.A.R.E. Cra 30 45-03, Bogota 111321, Colombia; l Department of Clinical Epidemiology & Biostatistics and Department of Medicine, McMaster University Health Sciences Centre, McMaster University, 1280 Main Street West, Room HSC-2C12, Hamilton, Ontario L8S 4K1, Canada; m Swedish Agency for Health Technology Assessment and Assessment of Social Services (SBU), S:t Eriksgatan 117, Stockholm SE-102 33, Sweden; n Department of Learning, Informatics, Management and Ethics, Karolinska Institutet, Tomtebodavägen 18 A, Stockholm SE-171 77, Sweden; o Bruyère Research Institute, University of Ottawa, 85 Primrose Avenue, Room 312, Ottawa, Ontario K1R 7G5; p MRC/CSO Social & Public Health Sciences Unit, University of Glasgow, Glasgow, Scotland, UK; q Department of Emergency Medicine, Cumming School of Medicine, University of Calgary, Calgary, Alberta; r Faculty of Medicine, Chiang Mai University, Chiang Mai, Thailand; s Cochrane Germany, Medical Center – University of Freiburg, Faculty of Medicine, University of Freiburg, Breisacher Strasse 153, Freiburg 79110, Germany; t Centre de Recherche Épidémiologie et Statistique Sorbonne Paris Cité – U1153, Inserm / Université Paris Descartes, Cochrane France, Hôpital Hôtel-Dieu, 1, place du Parvis Notre-Dame, Paris 75004, France; u NHMRC Clinical Trials Centre, The University of Sydney, Medical Foundation Building Level 6, 92-94 Parramatta Road, Camperdown, NSW 2050, Australia; v Knowledge Centre for the Health Services, Norwegian Institute of Public Health, PO Box 4404, Nydalen, N-0403 Oslo, Norway; w Mayo Clinic Evidence-Based Practice Center, Mayo Clinic, 200 1st Street SW, Rochester, MN 55905, USA; x Bruyère Research Institute & University of Ottawa, 85 Primrose Avenue, Ottawa, Ontario K1R 7G5; y Department of Health Research Methods, Evidence and Impact, McMaster University Health Sciences Centre, 1280 Main Street West, Hamilton, Ontario L8S 4K1, Canada; z Department of Medicine, McMaster University, Hamilton, Ontario, Canada; aa Division of Gastroenterology, Northwell Health/Hofstra University School of Medicine, Long Island Jewish Medical Center, Research Building B202, 270-05 76th Avenue, New Hyde Park, NY 11040, USA; ab Bruyère Research Institute and Ottawa Hospital Research Institute, University of Ottawa, Ottawa, Canada; ac Medicine Service, Birmingham Veterans Affairs Medical Center, 700, 19th Street South, Birmingham, AL 35233, USA; ad Department of Medicine at School of Medicine and the Division of Epidemiology at School of Public Health, University of Alabama at Birmingham, 1720 Second Avenue South, Birmingham, AL 35294-0022, USA; ae Department of Orthopedic Surgery, Mayo Clinic College of Medicine, 200 1st Street SW, Rochester, MN 55905, USA; af Iberoamerican Cochrane Centre, Biomedical Research Institute Sant Pau, CIBER of Epidemiology and Public Health (CIBERESP—IIB Sant Pau), Barcelona, Spain; ag Institute of Technology, University of Washington, 1900 Commerce St., Tacoma, WA 98402, USA; ah Public Health Department – Universidade Federal de Ciencias da Saude de Porto Alegre (Ufcspa), Rua Sarmento Leite, 245, CEP – CEP 90050-170 and HTA of Conceicao Hospital, Porto Alegre, Brazil; ai Department of Health Research Methods, Evidence, and Impact, McMaster University, 1280 Main Street West, Hamilton, Ontario L8S 4K1, Canada; aj Institute of Social and Preventive Medicine, University of Bern, Niesenweg 6, Bern 3012, Switzerland; ak IHCAI Foundation & Cochrane Central America & Spanish speaking Caribbean, Av 7. calles 35 y 37, No 35 30, Codigo Postal 10106, San Jose, Costa Rica; al Clinical Pharmacology Unit, Department of Medicine, Karolinska Institutet, Karolinska University Hospital, SE-171 76 Stockholm, Sweden; am Laboratory Medicine, Hôpital La Chartreuse, avenue Caylet, F-12200, Villefranche-de-Rouergue, France; an Department of Medicine, Faculty of Medicine, University of Ottawa, K1H 8M5, Ottawa, Canada

**Keywords:** Health, equity, GRADE, Guidelines, Systematic review, Meta-analysis, Subgroup analysis, Applicability, Indirectness

## Abstract

**Objectives:**

The aim of this paper is to describe a conceptual framework for how to consider health equity in the Grading Recommendations Assessment and Development Evidence (GRADE) guideline development process.

**Study Design and Setting:**

Consensus-based guidance developed by the GRADE working group members and other methodologists.

**Results:**

We developed consensus-based guidance to help address health equity when rating the certainty of synthesized evidence (i.e., quality of evidence). When health inequity is determined to be a concern by stakeholders, we propose five methods for explicitly assessing health equity: (1) include health equity as an outcome; (2) consider patient-important outcomes relevant to health equity; (3) assess differences in the relative effect size of the treatment; (4) assess differences in baseline risk and the differing impacts on absolute effects; and (5) assess indirectness of evidence to disadvantaged populations and/or settings.

**Conclusion:**

The most important priority for research on health inequity and guidelines is to identify and document examples where health equity has been considered explicitly in guidelines. Although there is a weak scientific evidence base for assessing health equity, this should not discourage the explicit consideration of how guidelines and recommendations affect the most vulnerable members of society.

**What is new?**

**Key findings**
• This paper provides consensus-based guidance for including health equity considerations in guideline development.

**What this study adds to what was known?**
• This paper adds an equity framework to the Grading Recommendations Assessment and Development Evidence (GRADE) guidance for rating the certainty of evidence in systematic reviews.

**What is the implication and what should change now?**
• Considering health equity in rating the certainty in synthesized evidence requires a *priori* elaboration of the disadvantaged populations and settings of interest, and methods to assess both relative and absolute effects for these populations.
• GRADE judgements about directness require transparent reporting of how judgements were made.

## 1 Background

Health inequities are differences in health that are not only unnecessary and avoidable but are also considered unfair and unjust [1]. As described in the introductory paper in this series, we use the acronym PROGRESS Plus (Place of residence, Race/ethnicity/culture/language, Occupation, Gender/sex, Religion, Education, Socioeconomic status, or Social capital + personal, relational and time-dependent characteristics) to identify individual and context-specific characteristics across which health inequities may occur [2].

Guideline panels need to decide early on whether they plan to develop equity-sensitive recommendations (as described in the introductory paper in this series). Using explicit prompts may be helpful in this process [3]. In principle, considering health equity is important for two main types of guidelines: (1) universal interventions where health inequity is a concern [4], [5], [6], [7]; and (2) targeted or dedicated interventions aimed at one or more disadvantaged populations that have experienced health inequities. An example of the latter is the Canadian immigrant health guidelines [8], developed to raise awareness of migrant health needs and improve access to effective preventive screening.

This paper provides guidance to address health equity when rating the certainty in synthesized evidence using the Grading Recommendations Assessment and Development Evidence (GRADE) approach. This paper is the third paper in a four-part series on health equity and GRADE, with the introduction [Welch et al.], overall process [Akl et al.], and evidence to decision methods [Pottie et al.].

## 2 Existing guidance

As discussed in the earlier two papers in this series, several authors have assessed how guidelines consider health inequity concerns [Welch et al. this series, Akl et al. this series]. None of these papers focus on rating the certainty of synthesized evidence (i.e., quality of evidence) using the GRADE approach.

## 3 GRADE certainty in synthesized evidence and health equity

The GRADE approach of presenting the evidence by outcome and the associated certainty (i.e., quality of evidence) involves the production of summary tables. These tables include evidence profiles (with details on the rating of certainty for each outcome) and summary of finding (SoF) tables that are intended for the public, patients, purchasers, payers, practitioners, product makers (e.g., manufacturers, industry), and policy makers [9].

Five methods can be used to assess health equity with the GRADE approach:

a) Include health equity as an outcome
b) Consider patient-important outcomes relevant to health equity
c) Assess differences in the magnitude of effect in relative terms between disadvantaged and more advantaged individuals or populations
d) Assess differences in baseline risk and hence the differing impacts on absolute effects for disadvantaged individuals or populations
e) Assess indirectness of evidence to disadvantaged populations and/or settings.

### 3.1 Consider including health equity as an outcome for the SoF tables

If health inequity is considered an important concern by relevant stakeholders, then health equity could be included as an outcome in the Population, Intervention, Comparison, Outcome questions, analytic framework, and SoF table. In doing so, guideline developers must recognize that health equity is primarily assessed with a subgroup analysis. The developers should also note that this may risk excluding other patient-important outcomes, if SoF tables are limited to only seven outcomes as recommended by GRADE. For example, the NICE guideline on maternal and child nutrition identified impact on health inequalities as one of its key priorities and framed its key question as: “What nutritional interventions are effective in improving the health of preconceptual, pregnant, and postpartum mothers and children (up to 5 years) and reducing nutrition-related health inequalities” [10]. By including health equity as an outcome in the SoF table, it is easier for guideline panels to find the information (or lack thereof) about health equity and consider it in their deliberations.

The direction and size of the effect on health equity is influenced by decisions such as the reference comparator group, use of relative or absolute measures, and whether the outcome is a desirable or undesirable event [11], [12]. For example, the choice of absolute or relative effects can change the conclusions about health inequalities. This is illustrated by gender disparity in stomach cancer mortality rates in the United States between 1930 and 2000 has decreased when looking at absolute differences (the rates for both men and women have declined). However, the relative risk for men compared to women has increased (increased disparity, male/female ratio) [13].

A lack of evidence about a critical health equity outcome should not be a reason to omit this from the SOF table. Indeed, this should be explicitly identified as an empty row, highlighting the need for further research to answer questions about health equity.

#### 3.1.1 Example 1

The Community Guide Water fluoridation guideline [14] included “health disparities” as an outcome in the analytic framework and the SoF table because the Community Task Force placed a high value on reducing socioeconomic disparities in dental caries. Socioeconomic disparities were measured as the difference in absolute terms of a continuous outcome (caries). The evidence review found three studies that provided insufficient evidence about socioeconomic disparities to draw conclusions, highlighting a gap in the evidence base (Table 1).

**Table 1:** Effect of Community Water Fluoridation on socioeconomic health inequities in caries [14]

Outcome	Measure	Evidence
Health equity as measured by socioeconomic disparities in caries	% of caries reduction	Inconsistent results on socioeconomic disparities (three studies)
	dmft/DMFT	No data on socioeconomic disparities

*Abbreviation:* DMFT/dmft, decayed, missing, or filled teeth.

Upper case refers to permanent teeth; lower case to primary teeth.

#### 3.1.2 Example 2

“Equity impact” was the primary outcome of a systematic review on interventions to reduce smoking in adults [15]. Equity impact was assessed as the difference in the magnitude of a dichotomous outcome in absolute terms, defined as a difference in absolute effect on prevalence in lower socioeconomic status compared to higher socioeconomic status. This review showed that while increases in price or taxes reduced health inequities in smoking, mass media campaigns were more likely to worsen health inequities. This type of review provides evidence that could be used to include impact on health equity as an outcome of interventions.

### 3.2 Consider patient-important outcomes relevant to health equity

As described in the previous paper in our series [Akl et al. in this series], the evidence synthesis process should consider the relative importance of different outcomes, determined with input from stakeholders representing disadvantaged groups. The evidence base for these outcomes should then be assessed. Examples of patient importance and health equity were provided in the previous paper in this series such as the importance of inconvenience of a subcutaneous chelation pump for people with sickle cell disease [described in Akl et al. in this series].

### 3.3 Assess differences in the magnitude of effect in relative terms between disadvantaged and more advantaged individuals or populations

Average effects obscure differences between subpopulations—that is, subgroup effects may exist. Examining whether effects differ across socioeconomic status or other variables relating to health inequity requires investigating heterogeneity in the treatment effect—for example, using statistical approaches such as meta-regression or subgroup analysis. However, such results may not be available in the literature. There is evidence that systematic reviews underreport subgroup analyses from primary studies [16], [17]. Furthermore, many primary studies fail to assess possible subgroup effects related to disadvantaged populations.

Relative effects are usually similar across diverse populations and settings, and spurious subgroup effects are common [18]. Thus, if analysis suggests an apparent subgroup effect, it is important to assess the credibility of the apparent effect [19]. Sun and colleagues [20], [21] describe several criteria to help do this such as determining a priori which subgroup analysis to conduct, finding a low *P*-value associated with a statistical test for interaction, and providing results from within-study comparisons. Sun et al. also showed that subgroup analyses reported in the literature rarely meet these criteria. Evidence synthesis that involves subgroup analyses should therefore consider the full set of credibility issues, using an appropriate checklist, and avoid making conclusions based on chance findings (Table 2).

**Table 2:** Checklist for assessing credibility of subgroup analyses [22]

Design Is the subgroup variable a characteristic measured at baseline or after randomization? Is the effect suggested by comparisons within rather than between studies? Was the hypothesis specified a priori? Was the direction of the subgroup effect specified a priori? Was the subgroup effect one of a small number of hypothesized effects tested? Analysis Does the interaction test suggest a low likelihood that chance explains the apparent subgroup effect? Is the significant subgroup effect independent? Context Is the size of the subgroup effect large? Is the interaction consistent across studies? Is the interaction consistent across closely related outcomes within the study? Is there indirect evidence that supports the hypothesized interaction (biological rationale)?

If applying the criteria in Table 2 leads to a conclusion that the subgroup effect is credible, the guideline panel should provide different estimates of relative and absolute effect for the subgroups. The panel should then consider making different recommendations for patients in these subgroups or consider whether recommendations that apply to the overall population need to be adapted to enhance equity. When the credibility of subgroup effects is low, the guideline panel may suggest that further research is needed. Few subgroup analyses meet all of these criteria; however, when most criteria are met, decision making must consider the likely existence of subgroup effects.

#### 3.3.1 Example: hypertension and ethnicity

The Eighth Joint National Committee guideline on management of hypertension recommends a calcium channel blocker or thiazide-type diuretic as initial therapy in the black hypertensive population (whereas an angiotensin-converting enzyme inhibitor, angiotensin receptor blocker, calcium channel blocker, or thiazide-type diuretic is recommended for others with hypertension) [23]. This recommendation was based on a prespecified subgroup analysis of the ALLHAT trial (*n* = 18,102 participants, 35% black [24]) that showed stroke was 51% (95% CI: 1.22, 1.86) greater for blacks treated with an ACE inhibitor first compared to those treated with a calcium channel blocker. The guideline panel rated this subgroup effect as moderate quality evidence. Had the panel not identified this subgroup effect, use of an ACE inhibitor as a first-line agent would have increased health disparities between black and white ethnic groups.

### 3.4 Assess differences in baseline risk and the differing impacts on absolute effects for disadvantaged individuals or populations

A higher baseline risk of adverse events in any population may lead to greater absolute harm from an intervention and conversely a higher baseline prevalence of the outcome of interest may lead to greater absolute benefit [25]. The SoF table should present the baseline risks and risk differences for each relevant population and provide supporting evidence. Because disadvantaged populations have a disproportionate burden of almost all health conditions, it is particularly important to consider the baseline risk for these populations. Baseline risk of adverse event rates or for the outcomes of interest for specific populations are best assessed using the most robust observational data on the actual population rather than from randomized trials. GRADE guidance regarding assessing certainty of estimates of risk from broad populations is available [26], [27].

#### 3.4.1 Example 1: WHO guidelines on vitamin A supplementation in children 6–59 months

In 2011, WHO recommended vitamin A supplementation for children aged 6 months to 5 years in countries where vitamin A deficiency is a public health problem (strong recommendation) [28]. This was based on findings of a Cochrane review with a relative risk for all-cause mortality of 0.76 (95% CI: 0.69, 0.83). The baseline risk of all-cause mortality was estimated at 0/1,000 in low-risk populations and 90/1,000 in high-risk populations (with vitamin A deficiency), based on control group event rates in the trials. Thus, the absolute effects in terms of numbers of deaths prevented with vitamin A compared to the control group were 0/1,000 for low-risk and 22/1,000 for high-risk populations.

#### 3.4.2 Example 2: national guide to a preventive health assessment for Aboriginal and Torres Strait Islander people

In Australia, a guideline panel sought to determine the optimal age at which to begin a series of preventive interventions in the Australian Aboriginal and Torres Strait Islander population. The panel recommended preventive interventions at an earlier age than the general population on the basis of higher prevalence of preventable diseases in Aboriginal and Torres Strait Islander populations. For example, type II diabetes is 3–4 times more common than in the general Australian population at all ages, leading to a recommendation for screening starting from age 18, instead of age 40 years for the general population [29].

### 3.5 Assess indirectness of evidence to disadvantaged populations

GRADE quality (or certainty) “reflects our confidence that the estimates of the effect are correct. In the context of recommendations, quality reflects our confidence that the effect estimates are adequate to support a particular recommendation. ‘Quality’ as used in GRADE means more than risk of bias and so may also be compromised by imprecision, inconsistency, indirectness of study results, and publication bias” [30]. Qualitative evidence may also be important when considering health equity. Certainty for qualitative evidence synthesis can be rated using the CerQUAL tool [31] in which the domain “relevance” is most closely aligned with directness.

Indirectness refers to the comparability between the population, the intervention, or the outcomes measured in research studies and those under consideration in a guideline or systematic review [32]. The GRADE approach evaluates the lack of directness as “indirectness.” Direct evidence may be lacking because some populations may not represent a large proportion of trial populations (e.g., migrants and refugees), and data are unlikely to be disaggregated for specific subgroups. Direct evidence may also be lacking because some populations are explicitly excluded from trials, such as pregnant women and people with multiple morbidities [33], [34], [35], [36]. Because multiple morbidities are more common in socioeconomically disadvantaged people [37], this may result in disproportionate exclusion of disadvantaged populations from trials. When direct evidence for the relevant disadvantaged population is not available, guideline developers will have to evaluate the indirectness of evidence obtained from other populations [38].

As a rule, certainty of the evidence should not be rated down for indirectness for population differences unless there are compelling reasons to anticipate differences in effect due to biology/physiology, sociocultural influences, or setting-specific resource issues that impact the effectiveness or harms of the intervention. In other words, one anticipates a different subgroup effect in either relative or absolute impact of treatment, though evidence is not available to make a formal assessment. (If it were, it should be formally assessed, as in Sections 3.3 and 3.4) Guideline panels need to consider that rating down for indirectness could in itself increase inequities if this leads to less use of an effective intervention by disadvantaged groups. In other words, lower certainty in effect estimates may lead to a weak recommendation and therefore under-use of a beneficial treatment. Rating down for indirectness should therefore be done cautiously because effective interventions are needed even more in some populations that are often excluded from trials, such as those with multiple morbidities.

#### 3.5.1 Example 1: Canadian migrant guidelines not rated down for indirectness

The quality of the evidence was not rated down for indirectness in the Canadian migrant guideline addressing screening for latent TB; the panel considered the evidence not to be indirect for migrants. Although no migrants were included in studies of intervention effectiveness, the developers did not expect different relative effects [39].

#### 3.5.2 Example 2: CDC guidelines for brief alcohol counseling for people with HCV infection rated down for indirectness

The Centers for Disease Control and Prevention recommended brief alcohol screening and counseling for all person with HCV infection, based on a systematic review of 22 randomized trials which found a reduction of alcohol consumption of 38.42% (95% CI: 30.91, 65.44) more than the control groups after 1 year. This evidence was rated down for indirectness by the guideline panel because none of the trials included persons with hepatitis C virus (HCV) infection [40].

## 4 Methodologic challenges

In developing this guidance, we identified a number of methodologic challenges. First, assessing effects on health equity is not a linear process. There may be a need to revisit the focus of the guideline during the evidence review process, including the consideration of important disadvantaged groups. NICE does this explicitly by revisiting their key questions regarding health equity throughout the process.

Second, there are often limitations in the underlying evidence base including poor reporting of sociodemographic characteristics [41], [42], under-reporting of subgroup analyses that are not statistically significant [21], [42], and use of multivariable models that may be overadjusted for effect mediators, and/or include unnecessary collinear variables [43]. Lack of evidence on whether the effects are consistent or different for disadvantaged populations makes it difficult to judge indirectness and rate certainty of evidence. When the evidence base is insufficient to assess effects on health equity, guideline panels need to make these limitations explicit and transparently report how they made judgments.

Third, epidemiologic evidence addressing baseline risk for specific disadvantaged groups may be difficult to obtain for the population or geographic region for which the recommendations are being developed. Health systems at local, regional, and national levels do not have consistent or reliable methods for reporting health status across all sociodemographic indicators of interest. Guideline panels should transparently report how they determined baseline risk estimates.

Fourth, assessing directness of evidence depends on the clinical and methodological expertise and judgment of SoF developers. The GRADE Guideline Development Tool includes an explicit checklist when producing SoFs to ask whether the evidence is direct across population, intervention, comparison, and outcome and document the decision for rating down, if performed.

## 5 Research agenda

The most important research priority in the field of health equity and guidelines is to systematically identify further examples of how guideline panels have assessed health equity considerations and incorporated these assessments into recommendations using transparent methods. For example, all WHO guidelines make their evidence to recommendation tables and SoFs publicly available for research such as this. These assessments could provide examples of whether, and how, the five issues (a–e) above have been considered for different situations, such as assessing the credibility of subgroup analyses and judging indirectness for disadvantaged populations.

In conclusion, the GRADE process provides a structured approach to assess effects on health equity. Health equity considerations warrant increased use of these methods in systematic reviews and guidelines. The findings of assessing health equity using these five steps in guideline development provides a basis for judging “impact on equity” which is part of the DECIDE framework, and details about this process are covered in the fourth paper of this series [Pottie et al.].
